# Frequency of sexual dysfunction in outpatients with severe mental illness in Greece

**DOI:** 10.3389/fpsyt.2023.1227218

**Published:** 2023-09-01

**Authors:** Maria Angelaki, Eirini Alexiou, Artemis Igoumenou, Giorgos Alevizopoulos

**Affiliations:** ^1^Department of Education, 251 Hellenic Air Force Hospital, Athens, Greece; ^2^Forensic Psychiatric Clinic, Sahlgrenska University Hospital, Gothenburg, Sweden; ^3^Center for Ethics, Law, and Mental Health (CELAM), Sahlgrenska Academy, University of Gothenburg, Gothenburg, Sweden; ^4^Division of Psychiatry, University College London, London, United Kingdom; ^5^Department of Psychiatry, Agioi Anargyroi Hospital, Faculty of Nursing, National and Kapodistrian University of Athens, Athens, Greece

**Keywords:** sexual function, sexual dysfunction, antipsychotics, schizophrenia, bipolar disorder

## Abstract

**Introduction:**

Patients with psychosis can develop sexual dysfunction, which may be related to the disease itself, psychosocial factors, somatic comorbidities, and the use of psychotropic medication.

**Objective:**

We aimed to investigate the type and frequency of sexual dysfunction in patients diagnosed with schizophrenia or bipolar disorder in order to assess the side effects of antipsychotics in sexual function.

**Methods:**

This is a multicenter, cross-sectional study, involving patients diagnosed with schizophrenia (79.3%) or bipolar disorder (20.7%) treated in the Department of Psychiatry and Community Mental Health Centers from November 2018 to December 2019. Patients were enrolled in the study after signed informed consent. Demographic and clinical data were collected from patients through a semi-structured interview. The Antipsychotics and Sexual Functioning Questionnaire (ASFQ) was administered to assess sexual function.

**Results:**

A total of 87 outpatients on antipsychotics were recruited in the study. The mean age was 43.6 years, while the mean duration of the disease was 16.9 years. Overall, only 9.1% of patients spontaneously reported sexual dysfunction. Patients treated with oral first-generation antipsychotics had more difficulties in achieving orgasm and decreased erection capacity. In contrast, patients treated with oral second-generation antipsychotics had decreased ejaculation capacity. Patients on antipsychotic combination therapy were associated with higher rates of sexual anhedonia.

**Discussion:**

These results suggest that sexual dysfunction is a side effect of antipsychotic treatment, which was spontaneously rarely reported by patients. It seems essential to obtain a psychosexual clinical history before initiating antipsychotic treatment to evaluate following changes and adopt an individualized strategy to manage sexual dysfunction induced by antipsychotics.

## 1. Introduction

The term “sexual dysfunction,” according to the 10th Revision of the International Statistical Classification of Diseases and Related Health Problems (ICD-10), covers the ways in which an individual is unable to participate in a sexual relationship as he or she would wish. Sexual response is a psychosomatic process and the etiology of sexual dysfunction usually involves both psychological and physical processes (1).

Sexual dysfunction in patients with psychiatric disorders may be associated with the psychiatric illness itself (negative symptoms, decreased initiative, and motivation), comorbid somatic diseases, psychosocial factors (stigmatization, discrimination), and the use of psychotropic drugs (2–6). Hence, it can be characterized as a multifaceted phenomenon underscoring the need for a better comprehension of the involved factors.

Regarding psychotropic medication, antidepressants, mood stabilizers, anxiolytic agents, and antipsychotics have been associated with sexual dysfunction related to drugs’ mode of action (7). Several studies have shown that different antipsychotics could disturb at least one of the three stages of the normal sexual response cycle comprising sexual desire (libido), sexual arousal (vaginal lubrication in women and erectile function in men), and orgasm (including ejaculatory function in men). The impact of the effects of antipsychotic medication on sexual function is related to the receptor binding profile of each agent (8–11).

Published studies suggest different effects between first-generation and second-generation antipsychotic drugs. Approximately 50% of patients with schizophrenia treated with first-generation antipsychotics report sexual dysfunction (8), while second-generation antipsychotics may have a better sexual side effect profile (12, 13). Mahmoud et al. showed that sexual function in patients diagnosed with schizophrenia was improved by switching from a first-generation to a second-generation agent compared to switching within the first-generation class of antipsychotics (12).

De Boer and colleagues, concluded to the following comparable order starting with antipsychotics that induce sexual dysfunction more frequently: risperidone, first-generation antipsychotics, clozapine, olanzapine, quetiapine, aripiprazole (14). However, in a meta-analysis found that quetiapine, ziprasidone, perphenazine, and aripiprazole were related to lower rates of sexual dysfunction (ranging from 16 to 27%), while olanzapine, risperidone, haloperidol, clozapine and thioridazine were related to higher rates of sexual dysfunction (ranging from 40 to 60%) (15).

The large heterogeneity of results in studies may be related to several factors, but mainly to the differences in both study design and psychometric instruments. Studies have shown that the incidence of sexual dysfunction was less than 10% when patients were asked about antipsychotic-related side effects and had the opportunity to report sexual dysfunction spontaneously. On the other hand, this rose to 30–60% in studies that have used structured interviews or self-report questionnaires (16–20).

Although antipsychotic-induced sexual dysfunction is rarely reported by patients with psychiatric disorders, it is estimated as one of the most distressing adverse effects of the treatment (21, 22). Subsequently, sexual dysfunction secondary to the use of antipsychotics may affect patients’ treatment, leading to decreased adherence to pharmacotherapy and reduced quality of life (10, 23, 24).

The presence and the severity of sexual dysfunction induced by antipsychotics is a prevalent matter in the daily life of a psychiatric patient. Thus, gaining knowledge on this subject is crucial to an improved clinical management. The authors have not come across any similar study among the Greek population of patients neither in the native nor in other languages.

The aim of this study was to evaluate the type and frequency of sexual dysfunction in outpatients suffering from schizophrenia or bipolar disorder treated with various antipsychotic agents. We used the Antipsychotics and Sexual Functioning Questionnaire (ASFQ), a validated instrument in the Greek population (25) that is used by researchers and clinicians in order to examine sexual side effects and to elucidate how antipsychotic medication relates to sexual function.

The main hypotheses of this study are: (1) sexual dysfunction is an underestimated side effect due to antipsychotic medication, as a minority of the patients will report spontaneously sexual disturbances, (2) different classes of antipsychotic medications induce different types of sexual dysfunction (3) first-generation antipsychotics and risperidone are associated with higher frequencies of sexual dysfunction than second-generation antipsychotics and (4) decreased sexual desire and erectile dysfunction are the most commonly reported sexual dysfunction.

## 2. Materials and methods

### 2.1. Study design

This was a multicenter cross-sectional naturalistic study. The participating clinical centers were the University Psychiatric Clinic of National and Kapodistrian University of Athens, the Greece Community Mental Health Center, and the Athens Community Mental Health Center. A convenience sample of 87 patients (45 females and 42 males) were recruited to participate in the study. Convenience sampling was applied due to the difficulty of finding a large number of patients willing to participate in a study concerning their sexual behavior.

Inclusion criteria for patients to this methodological study were:

1. Being between the ages of 18 and 50 years
2. Clinical diagnosis of schizophrenia or bipolar disorder according to the ICD-10 criteria
3. Oral and/or long-acting injectable antipsychotic treatment
4. Ability to understand and communicate in Greek
5. Ability to participate in the study, depending on their physical and psychological state, and
6. Informed written consent to participate in the study.

Exclusion criteria for patients in this study were:

1. Taking medication which, as shown in the package leaflet, causes sexual dysfunction at a frequency which is greater than 10%, except for antidepressants
2. Pre-existing diagnosis/intervention documented to induce sexual dysfunction
3. Alcohol abuse
4. Inability to give informed consent, and
5. Women in pregnancy or lactation.

A demographic data form and the Antipsychotics and Sexual Functioning Questionnaire (ASFQ) were administrated to all participants. They also completed the “Subjects’ Response to Antipsychotics (SRA)” Questionnaire in order to evaluate the validity of the ASFQ. Finally, they provided written informed consent. The period during which the study was conducted was from November 1st, 2018 to December 31st, 2019.

### 2.2. Ethical approval

The study was conducted with respect to the protection of participants’ rights, such as autonomy/self-disposition, privacy, anonymity-confidentiality, fair care, protection against harm, risk/benefit balance and informed consent. Specifically, the patients who recruited to this study signed an informed consent. In addition, the written permission to conduct the study was obtained from the Scientific Council of the Hospital, that outpatients visited. Ethical approval for the study was provided by the Human Rights and Ethics Committee of the Department of Nursing of the National and Kapodistrian University of Athens, Greece (National and Kapodistrian University of Athens, No. Prot. 170).

### 2.3. Assessments

The collection form of demographic, clinical, and other characteristics was created for the needs of this study by one of the researchers. The form included demographic data, such as age, gender, educational level, and social data including marital status, living status, presence of a current sexual partner, and employment status. Also, information about the patient’s health history, such as diagnosis, duration of the disorder, follow-up center, previous hospitalization in a psychiatric center, nicotine use, and history of suicide attempt, were included in the form.

The main outcome measure was the Antipsychotics and Sexual Functioning Questionnaire (ASFQ). The ASFQ is a questionnaire, which is completed during a semi-structured interview and is used to assess sexual function in patients receiving antipsychotics. Therefore, at the beginning of the semi-structured interview, the questions related to any previous antipsychotic treatment, the duration and the main reason for quitting previous antipsychotic treatment, the current antipsychotic treatment (antipsychotic and co-medication, including dosage), the experienced result/results of the treatment with the current medication treatment, as well as, the side effects mentioned spontaneously by the patient are recorded (25). Then, the ASFQ contains seven items for men and nine for women on sexual functioning. More specifically, it includes items about sexual desire (libido), orgasm, erection dysfunction, ejaculatory dysfunction, vaginal lubrication, and pain during intercourse (dyspareunia). It also includes items about amenorrhea, dysmenorrhea, galactorrhea, and gynecomastia. The questions about sexual function and any changes are structured to include a period of 4–6 weeks before the time of the interview. In order to ensure consistency in the structure of the ASFQ, each item is scored as 0 (unknown), 1 (significantly decreased), 2 (mildly decreased), 3 (unchanged), 4 (mildly increased), and finally, 5 (significantly increased) (20, 26). The ASFQ can be easily used by clinicians and researchers, as it guides them in introducing the topic in a nondirective and understandable way (25).

The ASFQ has been validated for the Greek psychiatric population and has demonstrated good psychometric properties. Specifically, it has shown exceptional internal reliability for research purposes, since Cronbach’s alpha coefficients were found to be between 0.90 and 0.94 for both sexes and good validity (25). The validity of the ASFQ for the 87 patients of the study was calculated by comparing the ASFQ with the corresponding items of the SRA.

The SRA is a 74-item self-report instrument that assesses patients’ responses to antipsychotic medication. It includes 8 subscales and answers are scored on a three-point scale: 0 (no), 1 (yes, to a certain degree), 2 (yes, to a high degree). The SRA includes 5 questions related to sexual activity: (1) I have more need for sex (item 38), (2) I have my periods less frequently (item 74), (3) I have less need for sex (item 17), (4) it’s more difficult to have an orgasm (item 55) and I have too little feeling for sex (item 70). The last three questions create the scale of sexual anhedonia, while the second question concerns only women. The time frame of interest of the SRA is the past week (27, 28).

### 2.4. Statistical analysis

IBM SPSS, V.21.0 (IBM Corp., 2012) was used to perform statistical analysis. Continuous variables are expressed as mean, standard deviation, median, minimum and maximum value, while categorical variables as numbers and percentages. Kolmogorov–Smirnov criterion (*p* > 0.05 for all variables) and normal probability plots were used to test the normality assumption.

The student’s t-test was used to assess the relationship between continuous variables and dichotomous variables. The Pearson’s correlation coefficient was used to assess the relationship between normally distributed continuous variables, and the Spearman’s correlation coefficient was used to assess the relationship between non-normally distributed continuous variables.

Demographics, clinical characteristics, and medication were the independent variables, while ASFQ sexual dysfunction scores were the dependent variables.

In case the dependent variable was a normally distributed continuous variable, and > 2 independent variables were statistically significant at the level of 0.2 (*p* < 0.2) in the bivariate analysis, multivariate linear regression was applied. The bilateral level of statistical significance was set equal to 0.05, so associations with *p* < 0.05 were considered statistically significant.

## 3. Results

### 3.1. Participant characteristics

The study included 87 patients, diagnosed with either schizophrenia (79.3%) or bipolar disorder (20.7%). The mean age was 43.6 years (SD 10.4), with a range of 19 to 65 years. The mean duration of the disease was 16.9 years (SD 8.9). The majority of patients were women (51.7%), single (66.7%) with a secondary level of education at least (71.3%). Furthermore, most of them were living with others (75.9%) and did not have a current sexual partner (67.8%). Detailed sociodemographic and clinical characteristics of the patients in the study are presented in Table 1.

**Table 1:** Demographic and clinical characteristics of the patients in the study.

Characteristics		*Ν*	%
Gender	Men	42	48.3
	Women	45	51.7
Age (years), mean (SD)		43.6 (10.4)	
Marital status	Married	15	17.2
	Single	58	66.7
	Divorced/separated, or widowed	14	16.1
Living status	Alone	21	24.1
	With others	66	75.9
Current sexual partner	No	59	67.8
	Yes	28	32.2
Level of education	Primary studies	25	28.7
	Secondary studies	36	41.4
	Higher education	26	29.9
Employment status	Unemployed	63	72.4
	Employed	24	27.6
Smoker tobacco	No	31	35.6
	Yes	56	64.4
Clinical center	Hospital	37	42.5
	Community Mental Health Center	50	57.5
Diagnosis	Schizophrenia	69	79.3
	Bipolar disorder	18	20.7
Duration of disorder (years), mean (SD)		16.9 (8.9)	
Previous hospitalization	No	27	31
	Yes	6	69
Suicidal attempt	No	73	83.9
	Yes	14	16.1

### 3.2. Medication treatment

The most common medications taken by patients were oral second-generation antipsychotics (74.7%), antidepressants [39.1%, (SSRIs: 25.3%)], antiepileptics (31%), long-acting injectable second-generation antipsychotics (29.9%), anxiolytics and hypnotics (29. 9%), anticholinergics (29.9%), and oral first-generation antipsychotics (24.1%). Table 2 shows the drug treatment received by the participants.

**Table 2:** The drug treatment received by the participants.

Drug treatment	*Ν*	%
Oral first-generation antipsychotics	21	24.1
Long-acting injectable first-generation antipsychotics	9	10.3
Oral second-generation antipsychotics	65	74.7
Long-acting injectable second-generation antipsychotics	26	29.9
Antidepressants (generally)	34	39.1
Antidepressants SSRIs	22	25.3
Antidepressants SNRIs	7	8.0
Tricyclic Antidepressants	5	5.7
Mood stabilizers	8	9.2
Anxiolytics	26	29.9
Antiepileptic drugs	27	31.0
Anticholinergic drugs	26	29.9
Antidiabetic drugs	2	2.3
Beta-adrenergic blocking agents	11	12.6
Antiparkinsonian drugs	1	1.1
Thyroid-hormone drugs	4	4.6
Lipid-lowering agents	3	3.4

At the time of enrollment, 59.8% were using oral antipsychotic medications, 21.8% were using oral and long-acting injectable antipsychotics and the rest (18.4%) were using only long-acting injectable antipsychotic medications. More thoroughly, regarding the oral treatment, the patients received the following first generation antipsychotics: trifluoroperazine hydrochloride (10–15 mg/day), haloperidol (10–25 mg/day), levomepromazine maleate (25–50 mg/day), pipaperone hydrochloride (40–120 mg/day), perphenazine (10–25 mg/day) and the following second-generation antipsychotics: clozapine (25–500 mg/day), risperidone (2–8 mg/day), quetiapine (150–350 mg/day), aripiprazole (15–30 mg/day), amisulpride (100–1,200 mg/day), paliperidone (3–9 mg/day), sertindole (4–16 mg/day), olanzapine (5–15 mg/day). Finally, regarding the long-acting injectable antipsychotics, the study patients received: haloperidol decanoate (50–200 mg/30 days), zuclopenthixol decanoate (200–400 mg/30 days), olanzapine pamoate monohydrate (210–405 mg/30 days), aripiprazole (400 mg/day), risperidone (50 mg/15 days), paliperidone (100–150 mg/30 days). The most common side effects reported by patients via the semi-structured interview are shown in Table 3. Only 9.1% of patients spontaneously reported sexual dysfunction.

**Table 3:** The most common side effects spontaneously reported by patients.

Adverse side effects	*Ν*	%
Fatigue	18	20.7
Sedation	20	23.0
Blurry vision	2	2.3
Dyslipidemia	1	1.1
Dry mouth	6	6.9
Mood swings	2	2.3
Akathisia	2	2.3
Nausea	2	2.3
Parkinsonism (tremor)	4	4.6
Dizziness	5	5.7
Sialorrhea	5	5.7
Tachycardia	4	4.6
Tardive dyskinesia	3	3.4
Hypertension	3	3.4
Bulimia	1	1.1
Decreased sexual desire	5	5.7
Decreased ejaculation volume	3	3.4
Numbness	2	2.3
Polyuria	1	1.1
Amenorrhea	2	2.3
Dermatitis	3	3.4
Weight gain	1	1.1
Memory disorders	1	1.1

### 3.3. Correlation coefficients between ASFQ and SRA

Patients’ sexual function was assessed using the Antipsychotics and Sexual Functioning Questionnaire (ASFQ) and the scale of sexual anhedonia of the Subjects’ Response to Antipsychotics (SRA) Questionnaire.

Cronbach’s alpha coefficients for ASFQ were 0.75 for men and 0.88 for women, indicating excellent ASFQ reliability. In addition, Cronbach’s alpha coefficient for the scale of sexual anhedonia of the SRA was 0.70, which indicates acceptable reliability.

The Spearman correlation coefficients between ASFQ and SRA are shown in Table 4. All correlation coefficients between SRA and ASFQ sexual dysfunction questions were moderately positive to strongly positive (between 0.28 and 0.55), indicating moderate to high validity of ASFQ. The same result was obtained for the correlation between the question “I have my periods less frequently” (item 74) in the SRA and the questions of sexual dysfunction in the ASFQ with the correlation coefficients having higher values (between 0.4 and 0.6). The results are similar to the question “I have more need for sex” (item 38) in the SRA with the correlation coefficients being negative this time. Negative correlations were expected, due to the reverse meaning of the instrument “SRA,” which shows that the higher the ASFQ levels the lower the SRA levels and *vice-versa*.

**Table 4:** The Spearman correlation coefficients (ρ) between ASFQ and SRA.

ASFQ	SRA					
	Sexual anhedonia		I have more need for sex		I have my periods less frequently	
	ρ	*p* value	ρ	*p* value	ρ	*p* value
Sexual desire	0.28	**0.010**	−0.37	**0.001**	0.50	**0.006**
Orgasm	0.40	**0.001**	−0.35	**0.003**	0.60	**<0.001**
Erection	0.12	0.500	−0.10	0.700	NA	NA
Ejaculation	0.45	**0.010**	0.06	0.800	NA	NA
Lubrication	0.55	**0.002**	−0.50	**0.009**	0.40	**0.040**
Pain during sexual intercourse	0.32	0.400	−0.55	0.100	NC	NC
Total sexual dysfunction of men	0.31	0.060	−0.30	0.060	NA	NA
Total sexual dysfunction of women	0.45	**0.004**	−0.40	**0.020**	0.50	**<0.001**

^*^ASFQ, antipsychotics and sexual functioning questionnaire; SRA, subject’s response to antipsychotics; NC, not calculated due to the limited variability; NA, not applicable to men. *p*-value <0.05 is considered statistically significant difference.

### 3.4. Sexual function

The patients’ sexual function is shown in Table 5. The scores are reversed; hence the higher mean values indicate worse sexual function. Thus, 52.6% of patients had decreased sexual desire and 46.6% had decreased orgasm. Also, 10.5 and 17.1% of patients reported galactorrhea and gynecomastia, respectively.

**Table 5:** ASFQ scores by patients of the study.

	*Ν*	%	Mean	(SD)	Median
Sexual desire			3.45	(1.27)	4.0
**Have you noticed a change in sexual desire since using the current antipsychotic?**					
Significantly decreased	20	26.3			
Mildly decreased	20	26.3			
Unchanged	21	27.6			
Mildly increased	7	9.2			
Significantly increased	8	10.5			
Orgasm			3.47	(1.07)	3.0
**Has your ability to achieve orgasm (come) changed since using the current antipsychotic**					
Significantly decreased	14	19.2			
Mildly decreased	20	27.4			
Unchanged	29	39.7			
Mildly increased	6	8.2			
Significantly increased	4	5.5			
Galactorrhoea					
**In the past four to 6 weeks, did milk leak from your breasts/nipples?**					
Yes	9	10.5			
No	77	89.5			
Swelling of breasts					
**In the last four to 6 weeks, did you notice a swelling of your breasts and/or nipples?**					
Yes	14	17.1			
No	68	82.9			
Erection			3.43	(0.88)	3.0
**Has your ability to have an erection changed since using the current antipsychotic?**					
Significantly decreased	5	14.3			
Mildly decreased	8	22.9			
Unchanged	20	57.1			
Mildly increased	1	2.9			
Significantly increased	1	2.9			
Priapism	0	0			
Ejaculation			3.81	(1.18)	4.0
**Have you noticed a change in the volume of ejaculate (sperm) since using the current antipsychotic?**					
Significantly decreased	13	40.6			
Mildly decreased	5	15.6			
Unchanged	10	31.3			
Mildly increased	3	9.4			
Significantly increased	1	3.1			
Sexual intercourse					
**Did you have sexual intercourse with a partner in the last four to 6 weeks?**					
Yes	18	42.9			
No	24	57.1			
Contraception					
**Are you using hormonal contraception?**					
Yes	5	11.9			
No	37	88.1			
Menstruation					
**Was your menstruation absent over the past four to 6 weeks?**					
Yes	18	40.0			
No	27	60.0			
Lubrication			3.34	(1.29)	3.0
**Have you noticed a change in the amount of vaginal lubrication (wetness) when you are sexually aroused since using the current antipsychotic?**					
Significantly decreased	6	20.7			
Mildly decreased	6	27.6			
Unchanged	9	31.0			
Mildly increased	2	6.9			
Significantly increased	4	13.8			
Sexual intercourse					
**Did you have sexual intercourse with a partner in the last four to 6 weeks?**					
Yes	14	31.1			
No	31	68.9			
Pain during sexual intercourse			2.0	(0.47)	2.0
**Did you have pain during sexual intercourse and has this changed since you began to use the current antipsychotic?**					
Deterioration	1	10.0			
Unchanged	8	80.0			
Improvement	1	10.0			
Total sexual dysfunction of men			14.22	(3.28)	14.0
Total sexual dysfunction of women			13.39	(4.26)	13.3
Total score of SRAs’ scale of sexual anhedonia			3.13	(1.85)	3.0

^*^ASFQ, antipsychotics and sexual functioning questionnaire; SRA, subject’s response to antipsychotics.

Specifically, 37.2 and 56.2% of men had decreased erection and decreased ejaculation, respectively. 42.9% of male patients had sexual intercourse with a sexual partner in the last 4–6 weeks from the enrollment. In women, 11.9% used hormonal contraception, 40% did not menstruate, and 31.1% had sexual intercourse with a partner in the last 4–6 weeks from the time of enrollment. Also, 48.3% of women had decreased lubrication.

The mean scores of sexual desire, orgasm, erection, ejaculation, and lubrication were between 3 and 4, i.e., higher than the median point (=3), which indicates worse sexual function of patients. The same result emerges from the overall score of sexual dysfunctions in men and women, which were greater than the median point (=12.5).

The association between sexual dysfunction and the different variables analyzed using multivariant linear regression, shows that age, occupation, and educational status, correlate significantly to sexual dysfunction. Also, patients who received oral first-generation antipsychotics had decreased ability to achieve orgasm (*p* = 0.05) and decreased erection (*p* = 0.016), while those who received oral second-generation antipsychotics had decreased ejaculation (*p* = 0.017). A positive correlation between the number of various antipsychotics and sexual anhedonia was found (*p* = 0.019). There is also a positive correlation between the number of antipsychotics’ side effects and the sexual anhedonia (*p* < 0.001), and specifically the decreased ejaculation (*p* = 0.006). This data is shared in Table 6.

**Table 6:** Associated factors of sexual dysfunction by multivariant lineal regression.

		Multivariant linear regression		
		b-Coefficient	95% CI	*p* value
Sexual desire	Age	0.04	0.020 to 0.070	0.003
	Education	0.3	0.004 to 0.650	0.004
Erection	Education	0.5	0.200 to 0.800	0.003
	Oral first-generation antipsychotics	0.7	0.100 to 1.280	0.016
Vaginal lubrication	Age	0.05	0.009 to 0.820	0.016
Orgasm	Age	0.03	0.010 to 0.050	0.015
	Education	0.4	0.070 to 0.660	0.017
	Oral first-generation antipsychotics	0.6	0.001 to 1.190	0.05
	Unemployment	0.7	0.200 to 1.200	0.012
Ejaculation	Number of antipsychotics’ side effects	0.1	0.020 to 0.130	0.006
	Oral second-generation antipsychotics	0.9	0.200 to 1.600	0.017
Total sexual dysfunction	Age	0.12	0.040 to 0.200	0.003
	Education	1.2	0.300 to 2.200	0.011
Sexual anhedonia	Combined antipsychotic treatment	0.6	0.100 to 1.200	0.019
	Number of antipsychotics’ side effects	0.14	0.100 to 0.180	<0.001

## 4. Discussion

### 4.1. Main findings

The main finding of the present study was that different classes of antipsychotic medications induced different types of sexual dysfunction. Specifically, patients who received first-generation antipsychotics experienced mostly erectile dysfunction and difficulty in achieving orgasm, while participants who were treated with second-generation antipsychotics reported mainly ejaculatory problems. Also, antipsychotic polypharmacy might have a multiplicative effect.

Like other studies concerning the nature and the frequency of sexual dysfunction in antipsychotic treated outpatients with schizophrenia or bipolar disorder under regular mental health care (29–31), the present study supported that sexual dysfunction is an underestimated side effect due to antipsychotic medication. Only, a minority of the patients report spontaneously sexual disturbances. This fact may lead patients to significant consequences since sexual dysfunctions are among the main causes that lead patients to stop taking drugs (32). Generally, this finding illustrates the crucial role of the doctor-patient relationship. Indeed, studies have shown that the attachment style is an essential mediator of close relationships such as the therapeutic one between patients with bipolar and psychotic spectrum disorders and the physician and it is associated with treatment non-adherence (33).

Undoubtedly, there are several studies that have compared the impact on sexual functioning by first-generation and second-generation antipsychotics (12, 34–37) as well as studies that have compared the sexual adverse effects between different second-generation antipsychotic agents (29, 30, 38, 39). The majority of them conclude that first-generation antipsychotics and risperidone are associated with higher frequencies of sexual dysfunction, whereas second-generation antipsychotics, such as clozapine, olanzapine, quetiapine, and aripiprazole, are correlated with lower frequencies (12, 14, 22). Seeking an explanation for these findings, it seems that blockade of dopamine activity, hyperprolactinemia induced by antipsychotics, and alpha-1receptor blockade are strongly linked to treatment-induced sexual dysfunction in psychiatric patients (6, 14).

Investigating the impact of different categories of antipsychotic drugs on sexual function according to the three main phases of the normal cycle of sexual response, namely desire, arousal, and orgasm, we found that the rates of sexual dysfunction were high in each phase separately.

More than half of the patients participated this study had reduced sexual desire. These results are consistent with international data reporting decreased desire as the most commonly reported sexual dysfunction in both genders (11). However, a few patients mentioned improved sexual functioning over time, possibly related to an improvement in the symptoms of the psychotic disorder. It is difficult to estimate changes in sexual desire, as a psychiatric disorder itself can affect sexuality. Furthermore, the impact on the sexual desire caused by antipsychotic drugs cannot be described as well as the other phases of sexual dysfunction (39).

As far as the phase of sexual arousal, the rates of reduced erectile function and vaginal lubrication in our study seem to confirm those of previous studies (3, 11, 36). Erectile dysfunction, like impairment of sexual desire, is supposed to be also an equally common sexual side effect (3, 39). In the study of Nagaraj et al. erectile dysfunction was associated more with first-generation antipsychotics than with second-generation antipsychotics (36). The association of erectile dysfunction and the treatment with first-generation antipsychotics was also reported in the current study. Higher rates of sexual dysfunction in females in form of decreased vaginal lubrication can be explained in terms of the greater effect of antipsychotic medication on the prolactin levels of women than men (40).

In most published studies, desire and erection problems induced by antipsychotics were more reported than orgasmic and ejaculatory problems. A possible explanation may be the co-occurrence of erectile dysfunction, since a patient who cannot achieve a complete erection, he may think that he has not the ability to ejaculate (39). However, the present study found that the percentage of orgasmic dysfunction in patients treated with antipsychotics was extremely high. This finding seems to be in line with a recent study from Nigeria (41), which suggests that the most common problem reported by patients suffering from schizophrenia was the inability to achieve orgasm followed by satisfaction with orgasm.

Specifically, we found that the ejaculation problem was the most frequent sexual side effect followed by erection failure in males. Decreased ejaculation volume (DEV) was reported by more than half of the men and was linked to treatment with second-generation antipsychotics. However, it is noteworthy that orgasmic problems were rather linked to first-generation antipsychotics. According to studies, DEV is related to treatment with several antipsychotics, not only first-generation but also second-generation, but the mechanisms are poorly understood (14). Priapism was not reported by any of the patients in the study.

Although menstrual disturbances, gynecomastia, and galactorrhea are not included in sexual dysfunctions (14), they were assessed in this study because of their association with serum prolactin levels. Literature findings suggest the higher the prolactin level is, the more patients have clinical symptoms. These manifestations are partially due to the hypogonadism caused by prolactin, which disturbs hypothalamic–pituitary axis function. Thus, patients can suffer from sexual dysfunction, infertility, amenorrhea, gynecomastia, or galactorrhea. Nevertheless, in this study the majority of patients did not report gynecomastia and galactorrhea (42). The majority of patients did not report gynecomastia and galactorrhea.

In our study, antipsychotic polytherapy was associated with even higher prevalence rates of sexual dysfunction, which is consent with the recent literature (43). Although there are significant differences in the sexual dysfunction rates of antipsychotics, there is strong evidence that actually all of these agents can affect sexual function (15, 44). Hence, it makes sense to suggest that co-administration of many antipsychotics can result to a sum of their side effects.

### 4.2. Correlations between socio-demographic characteristics and sexual dysfunction

Correlations between socio-demographic characteristics and sexual dysfunction were found for age, employment status, and educational level. The majority of the patients in the study were current smokers. Although previous studies found that patients who smoke show a deterioration in sexual function (45), in this study nicotine use *via* smoking did not seem to be associated with sexual dysfunction.

Initially, the increase in age appeared to be associated with deterioration in all stages of sexual function, i.e., sexual desire, sexual arousal (reduced lubrication in women), and orgasm. Sexual impairment due to aging has been reported in studies carried out in the general population (46, 47), and patients diagnosed with schizophrenia (38, 48–50). Although some studies suggest the opposite (51), it is well known that this may be caused by the human adjustment to biological and physiological factors during the lifetime, which subsequently has effects on sexual activity.

Also, in our study, unemployed people had more difficulty in achieving orgasm than employed people. This finding could be explained by their quality of life. Quality of life can be defined as an individual’s perception of well-being and satisfaction with his or her living conditions including sexual functioning, as well as the health status and access to external resources, and opportunities, such as social support (52–54). Thus, quality of life is a factor influenced by socio-demographic characteristics (53, 55), psychiatric symptoms (53, 56), cognitive abilities (53), antipsychotic medication (12, 57, 58), and its side effects (59). Living conditions, such as living arrangements and employment status, may influence the quality of life of patients with psychiatric disorders (60).

Worth to mention that, our study found that the higher the educational level was, the more decreased sexual desire, decreased ability to achieve orgasm, and decreased erection there were. The association between educational level and sexual functioning for patients with severe mental disorders is still ambiguous (60). Explaining this relationship in the context of quality of life, some studies found that psychiatric patients with higher educational level report lower quality of life (61), whereas others suggest a positive correlation between education status and quality of life (62). These conflicting findings result from the combination of expectations, duration of disease, and interactions between employment, income, and level of education. In the study of (63), a negative correlation between the quality of life and educational level in low-income populations was found. Patients with a severe mental illness may have lower expectations due to their disorder itself and their poorer living conditions (60, 61).

### 4.3. Limitations

Several limitations should be considered when interpreting these results. The present study, like other studies on sexual function in schizophrenia (31, 38, 39), was cross-sectional. The cross-sectional design limits the strength of the causal relationship; hence it is impossible to study the cause-effect relationship between the antipsychotic treatment and the sexual dysfunction.

Another limitation was the non-random sampling. By applying non-random sampling, convenience samples were obtained, so it is not feasible to generalize the results of the study to the population-source from which the sample came. Non-randomization does not rule out the possibility that other factors except for antipsychotic treatment, such as other medical conditions like obesity, diabetes parkinsonism or comorbidity with substance use, highly common in individuals with severe mental illness, may affect the onset of sexual dysfunction during the study period. Interpersonal conflicts, common in individuals with severe mental illness, may also lead to sexual dysfunction.

A third limitation was the small sample size. Patients who, according to the treating psychiatrists, should not participate in the study, due to a new psychotic episode, relapse, or obsession with sexuality issues that would interfere with their treatment, were excluded. Thus, the sample size was too small to compare the possible effects of specific antipsychotic agents in a reliable way. It is worth noting that the availability of the antipsychotic agents at the place and time of the study plays an important role. At the time of the study, some antipsychotics were not available in Greece, while others were little used.

Finally, few patients in the sample received antipsychotic monotherapy. Most patients were receiving a variety of antipsychotic drugs while receiving other psychiatric and non-psychiatric medications, like antidepressants, anticholinergics, and antiepileptics in high percentage. Thus, it is difficult to distinguish if the sexual dysfunction was related to antipsychotics or polypharmacy.

Despite the above-mentioned limitations, the present study has several strengths. Although the non-random sampling of participants, the subjects were outpatients diagnosed with schizophrenia or bipolar disorder who reside in the community and visited public mental health settings. Also, the main psychometric instrument in the current study was the validated Greek version of the ASFQ, which has established psychometric characteristics (internal reliability and validity) in the assessment of the presence and severity of sexual dysfunction (25). Finally, it was the first study on the frequency of sexual dysfunction in outpatients with a psychotic disorder in Greece.

## 5. Conclusion

This study, given the lacking research in this field in a Greek concept, is the first to provide estimates of the incidence of sexual dysfunction in Greek outpatients with schizophrenia or bipolar disorder using the Greek version of the ASFQ. These results suggest that sexual dysfunction is one of the side effects of antipsychotic treatment, which was spontaneously rarely reported by patients. Additionally, different types of sexual dysfunction were induced among patients who receive first-generation antipsychotics and those who receive second-generation antipsychotics. Given the fact that many patients with severe mental illness will be on antipsychotic medications lifelong and even patients on relatively short antipsychotic treatment experience significant sexual dysfunction, it is important to understand the complex pathophysiology of the sexual dysfunction induced by antipsychotics seen among patients with severe mental illness and find pharmacological strategies to alleviate this distressing persistent side effect. Moreover, it seems essential to obtain a psychosexual clinical history before initiating antipsychotic treatment to evaluate following changes and adopt an individualized strategy to manage sexual dysfunction induced by antipsychotics. More research needs to be conducted on the impact of pharmacological choices on sexual function.

## Data availability statement

The raw data supporting the conclusions of this article will be made available by the authors, without undue reservation.

## Ethics statement

The studies involving humans were approved by Ethical approval for the study was provided by the Human Rights and Ethics Committee of the Department of Nursing of the National and Kapodistrian University of Athens, Greece (Athens, No. Prot. 170). In addition, the written permission to conduct the study was obtained from the Scientific Council of the Hospital, that outpatients visited. The studies were conducted in accordance with the local legislation and institutional requirements. The participants provided their written informed consent to participate in this study.

## Author contributions

MA and GA: conception and design. MA: data acquisition and analysis and interpretation of data. MA, EA, AI, and GA: drafting the manuscript. MA, EA, AI, and GA: revising the manuscript for intellectual content and final approval of the completed manuscript. All authors contributed to the article and approved the submitted version.

## Conflict of interest

The authors declare that the research was conducted in the absence of any commercial or financial relationships that could be construed as a potential conflict of interest.

## Publisher’s note

All claims expressed in this article are solely those of the authors and do not necessarily represent those of their affiliated organizations, or those of the publisher, the editors and the reviewers. Any product that may be evaluated in this article, or claim that may be made by its manufacturer, is not guaranteed or endorsed by the publisher.
